# Identification of relevant prognostic values of cytokeratin 20 and cytokeratin 7 expressions in lung cancer

**DOI:** 10.1042/BSR20171086

**Published:** 2017-11-06

**Authors:** Hai-Tao Luo, Cai-Xia Liang, Rong-Cheng Luo, Wei-Guang Gu

**Affiliations:** 1 Department of Oncology, Affiliated Hospital of South China Sea, Southern Medical University, Foshan 528200, P.R. China; 2 Department of Respiratory Medicine, Affiliated Hospital of South China Sea, Southern Medical University, Foshan 528200, P.R. China; 3 Department of Oncology, TCM-Integrated Hospital of Southern Medical University, Guangzhou 515000, P.R. China

**Keywords:** CK20, CK7, lung cancer, survival outcome

## Abstract

Lung cancer is one of the most common malignant tumors harmful to human health. Cytokeratin (CK) is highly conserved and differentiated related to the proliferation and differentiation of epithelial cells. The aim of the study was to explore expressions of CK20 and CK7 and corresponding prognostic values in patients with lung cancer. Our study included 258 cases of patients confirmed with lung cancer. Expressions of *CK20* and *CK7* mRNA and protein were detected using real-time quantitative PCR (qRT-PCR) and Western blot, respectively, followed by the performance of immunohistochemistry staining. Associations of CK20 and CK7 with the clinical parameters and prognosis of lung cancer patients were further analyzed. There were obvious differences regarding the positive expression of CK20 in different T stage, lymph node metastasis, invasion, size, and clinical stage subgroups; besides, significant differences in the positive expression of CK7 were also observed in subgroups of different sex, age, lymph node metastasis, invasion, and differentiation. Furthermore, effects of age, smoking, T stage, lymph node metastasis and invasion, size, and CK7 expressions were significant on the survival of patients (all *P*<0.05). Multivariate analysis revealed that lymph node metastasis, T stage, and CK7 expression were independent risk factors for poor prognosis of involved patients (all *P*<0.05), while age, smoking, and invasion had no marked relation to the survival time of patients with lung cancer (all *P*>0.05). Positive CK20 and CK7 expressions are detected in patients with lung cancer; positive expression of CK7 associated with pathological features of lymph node metastasis and T stage may be independent clinical parameters for poor prognosis of patients with lung cancer.

## Introduction

Lung cancer is one of the most common malignant tumors in the world that can be harmful to human health and life, despite significant improvements in diagnostic and therapeutic approaches to this malignancy [1]. For nearly half a century, the incidence and mortality of lung cancer have increased significantly [2]. According to statistics, total number of lung cancer cases account for 13% of all new malignant tumors diagnoses, over 1.6 million new cases were confirmed as lung cancer in 2008, causing approximately 1.4 million deaths accounting for 18% of all cancer deaths, ranking first worldwide [3]. With the development of the industrialization and continuously growing population in China, ageing intensifies and lung cancer relevant risk factors change, therefore, lung cancer has become one of the predominant causes for cancer-related deaths in recent years [3,4]. More than 80% of the patients have lost their best time for surgery and multidisciplinary radical cure when admitted to the hospital, overall 5-year survival rate of patients with lung cancer is therefore extremely low [5,6]. There can be no doubt that a comprehensive understanding of the epidemiology of lung cancer providing background and contextual information is important for management of guidelines in such cancer. Tobacco smoking is still the major cause of most types of lung cancers, making up over 90% of lung cancer related deaths [7]; occupational and environmental exposure, ionizing radiation, as well as previous chronic lung infection are also partially responsible for the prevalence of lung cancer [8–10]. More importantly, this cancer is also characterized by multiple genetic/epigenetic alterations, involving the activation of carcinogenic factors and inactivation of cancer suppressive factors. There is no question that a better understanding of the molecular mechanism by which these alterations affect lung cancer pathogenesis will provide new and more effective strategies for early diagnosis and targetted treatment regimens formulation.

Cell cytokeratin (CK) is a main cytoskeletal protein in the keratinocyte that is mainly distributed in the epithelial cells, maintaining the integrity and continuity of the epithelial tissue is the major function of such type of structural protein [11,12]. Studies have found that CK is highly conserved and differentiated, closely related to the proliferation and differentiation of epithelial cells [13]. At present, there are more than 20 kinds of confirmed subtypes distributed in epithelial cells of different organs [14]. As reported, the involvement of CK may be crucial for the development of human cancers. For example, CK5 could promote the progression of breast cancer by up-regulating transcriptional repressor activity [15]. CK 18 has also been investigated in breast cancer and was confirmed to play a diagnostic role in this cancer [16]. Amongst them, CK7 is distributed in parts of the bronchi, breast, and endometrial secretory cells, CK20 distribution is found in intestinal gland crypt cells and cutaneous Merkel cells [17,18]. Clinical studies revealed that CK20 expression might have an effective prediction role of overall survival in metastatic colorectal cancer patients who underwent chemotherapy treatment [19]; meanwhile, CK7 might predict the response to concommitant radiochemotherapy for locally advanced cervical cancer [20]. With respect to CK protein expression in lung adenocarcinoma, CK7/CK20 are suggested to possess certain characteristics of immunophenotype, critical for the differential diagnosis of pulmonary primary lung adenocarcinoma and other parts of the origin of metastatic adenocarcinoma [21]. Refining the understanding of the etiology and pathogenesis of lung cancer remain a vibrant area of research. In the present study, we therefore investigated the role of CK7 and CK20 expressions with clinical parameters and prognostic outcomes of lung cancer, so as to explore the association between CK7/CK20 and lung cancer, and to study the influence of various clinical parameters on the prognosis of lung cancer patients, thus making a meaningful exploration for lung cancer diagnosis, treatment, and prognosis.

## Materials and methods

### Ethics statement

This cohort study was approved by the Institutional Committee of Affiliated Hospital of South China Sea, Southern Medical University. Written informed consents were obtained from all the enrolled subjects and the study conformed to the guidelines and principles of Declaration of Helsinki.

### Subjects

The present study collected a total of 258 cases with clinically diagnosed lung cancer in Affiliated Hospital of South China Sea, Southern Medical University from January 2009 to December 2012, with complete clinical data and follow-up data integrity. All patients were admitted to the hospital and managed by surgical dissection. There were 103 female and 155 male cases. Enrolled patients’ ages centralized in 35–72 years, with a mean age 54.33 ± 10.41 years. A sum up of 64.73% (167/258) patients had smoking history. Clinical stages classification of lung cancer was achieved according to the seventh edition of the standard of tumor node metastasis (TNM) staging which was set by the International Union of Counter Cancer (UICC) in 1997 [22]. Corresponding clinical stages classification results were as follows: stage I of 143 cases; stage II of 60 cases; and stage III of 55 cases. All cancer tissues and adjacent normal tissues (paracancerous tissues, 5 cm near cancer tissues) were harvested during surgical process. Collected samples were divided into three parts equally and proportionately, one for RNA extraction to detect the relative mRNA expressions, another for the extraction of protein to detect the protein expressions, and the remaining specimens were fixed in neutral formalin solution, and were then transparent, dehydrated, paraffin embedded, and preserved for immunohistochemistry.

### Real-time quantitative PCR detection

Real-time quantitative PCR (qRT-PCR) assay was applied to detect the expression of CK7 and CK20 in adjacent normal tissues and lung cancer tissues. Approximately 100-mg frozen tissues were prepared for RNA extraction using miRNeasy Mini Kit (Qiagen, Germany). RNA concentration and purity were determined with the general absorbance at 260/280 nm (OD260/OD280) ratio between 1.7 and 2.1. Following reverse-transcriptase synthesis of the corresponding cDNA template, ABI7500 quantitative PCR (ABI, U.S.A.) was used for qRT-PCR amplification reaction, with the reaction system of 10 μl of 2× Allinone™ qPCR mix, 1 μl upstream and downstream primers, respectively, 1 μl reverse transcription cDNA, and ddH_2_O to 20 μl. Reaction conditions were: 95°C predenaturation for 10 min, denaturation for 10 s at 95°C, annealing for 20 s at 60°C, extending to 30 s at 72°C, with a total of 35 cycles. The primers used for reaction were: CK7: 5′-GTTCCATTTGCAAAGGCTGT-3′ and 5′-CAGGTGGTTACCCGAAAGA-3′; CK20: 5′-GGAAGTCGATGGCCTACACAA-3′ and 5′-GGCCTGGAGCAGCATCAA-3′; GAPDH: 5′-CCCATCACCATCTTCCAGG-3′ and 5′-CATCACGCCACAGTTTCCC-3′. GAPDH was taken as the internal reference (Shanghai JiMa Pharmaceutical Technology Limited Company, China); 2^−ΔΔ*C*_t_^ was used to reflect the relationship between the expression ratio of the target gene in the experimental group and the control group, amongst which *C*_t_ was the number of amplification cycles when the real-time fluorescence intensity of the reaction reached the set threshold. This experiment was repeated three times.

### Western blot detection

Western blot assay measured CK7 and CK20 expressions in adjacent normal tissues and lung cancer tissues. Following the extraction of protein from the tissue samples, the protein concentration was measured in strict accordance with the instructions on the BCA kit. Added with loading buffer (30 μg for each well) and boiled at 95°C for 10 min, the protein separated by PAGE (10% gel) was conducted with a tube voltage of 80–120 V. Both BCA kit and polyacrylamide gel were purchased from Boster Biotech Co. Ltd (Wuhan, China). Wet transfer was run on PVDF membrane under 100 mV for 45–70 min. Then, the reaction was blocked with 5% BSA at 37°C for 1 h. Primary antibodies of CK7 and CK20 (dilution ratio of 1:1000) were added and incubated overnight at 4°C. After three washes with TBS plus Tween buffer (5 min per time), membranes were incubated with corresponding secondary antibodies at room temperature for 1 h and rinsed for three times again. With β-actin as an internal reference, chemiluminescence reagent and Gel Doc EZ imager (Bio–Rad Laboratories, California, U.S.A.) were used for development. As for the semiquantitative analysis of Western blot results, GeneTool software was applied to determine the protein expression levels of CK7 and CK20 according to the ratio of integral values of optical density of the target proteins (CK7 and CK20) and reference (β-actin). The experiment was repeated three times.

### Immunohistochemistry staining

All the specimens were fixed with 10% formalin for 24 h and embedded with paraffin. The wax block was sliced 5-μm thick each for four consecutive slices. The known positive-stained histological section was used as the positive control. PBS buffer instead of the primary antibody was regarded as the negative control. Paraffin sections were dewaxed after hydration, with PBS (pH: 7.4), rinsed for three times, each time for 3 min. A drop each or 50 μl peroxidase blocking solution was added on each slice to block the endogenous peroxidase activity, and then incubated for 10 min at room temperature. Next, samples were rinsed in PBS for three times, 3 min each time. After removing PBS solution, a drop or 50 μl of non-immune animal serum was added in each slice, following room temperature incubation for 10 min. And then, the serum was removed, and a drop or 50 μl of the first antibody (rabbit anti-human CK7 monoclonal antibody and rabbit anti-human CK20 monoclonal antibody, purchased from Wuhan Boster Biological Engineering Co., Ltd.) was added into each section, and incubated at room temperature for 60 min, followed by incubation at 4°C overnight. Afterward, sections were washed with PBS again for another three times, each time for 5 min. After removing the PBS liquid, a drop per slice or 50 μl biotin-labeled second antibody was supplemented at the section, which was then placed at room temperature for incubation (10 min). Subsequently, following another three times of PBS rinsing (3 min each) and the removal of PBS liquid, a drop or 50 μl streptomycin-avidin peroxidase solution was added into each slice; treated sections were then incubated at room temperature for 10 min. The section was rinsed in tap water, followed by Hematoxylin staining (Beijing Chemical Factory), 0.1% HCl was used as differentiation liquid for differentiation, and ammonia solution or 0.1% PBS washing for blue staining. At last, after DAB staining, tissue blocks were dehydrated in ascending series of alcohol, cleared in xylene, and embedded in paraffin.

### Result interpretation

The Leica pathological image analysis instrument (QMR+Q550) was used for the analysis of the test results under observation. CK20 and CK7 were located in the cytoplasm, positive expression was identified as brown or yellow staining of the cytoplasm or nucleus, and 400 times (×400) magnification were observed in ten fields of vision. The percentage of positive cells score was described as follows: positive cell percentage of less than 1%, 0 point; 1–25%, 1 point; 25–50%, 2 points; 51–75% for 3 points; and over 75% for 4 points. Scoring was further counted according to the cell staining intensity score: colorless, 0 point; light yellow, 1 point; brown yellow, 2 points; and deep yellow, 3 points. The product of the above two scores for 0 point was regarded as negative (−); 1–2 points, positive (++); and >2 points, strongly positive (+++). In order to facilitate statistical analysis, the negative case was defined as the negative group, the positive and strong positive cases were defined as the positive group. Results were determined with double-blind method.

### Follow-up

A 10–60 months follow-up study was conducted in 258 lung cancer patients. Follow-up analysis was made based on outpatient service revisit and telephone. Survival outcomes were recorded and measured for further analysis between groups.

### Statistical analysis

SPSS18.0 software and GraphPad Prism6 were used for data analysis. Relative mRNA expression and protein expression levels were expressed as mean ± S.D., homogeneity test of variance was first performed for each group, and then *t* test was used for the comparison between groups. χ^2^ test was performed for data correlation analysis, the cumulative 5-year survival rates were calculated by the life-table method, patients’ overall survival curve within the period was drawn by using Kaplan–Meier methods to determine the association of detected factors and patients’ survival, and COX proportional hazards regression model was used for exploring the relation between variables and prognosis. *P*<0.05 indicated significant differences.

## Results

### *CK7* and *CK20* mRNA expressions detected by qRT-PCR detection

**Figure 1:**
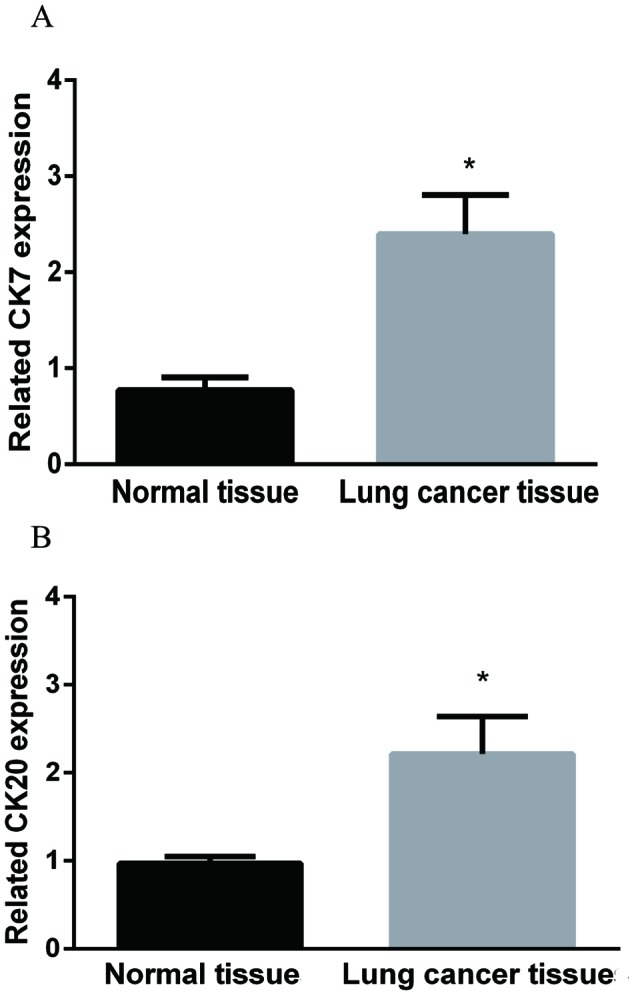
qRT-PCR detection results about the relative mRNA expressions of CK7 and CK20 in lung cancer and paracancerous normal tissues (**A**) CK7 relative mRNA expression in lung cancer tissue was significantly higher than that in paracancerous normal tissues. (**B**) CK20 relative mRNA expression was also higher in lung cancer tissue when compared with that in paracancerous normal tissue. *, *P*<0.05, the difference between groups was significant.

Detection results of mRNA expressions of CK7 and CK20 in lung cancer and paracancerous tissues showed that both CK7 and CK20 were differently expressed, significantly higher in lung cancer tissues than those in paracancerous normal tissues (both *P*<0.05), as shown in Figure 1.

### CK7 and CK20 protein expressions detected by Western blot

**Figure 2:**
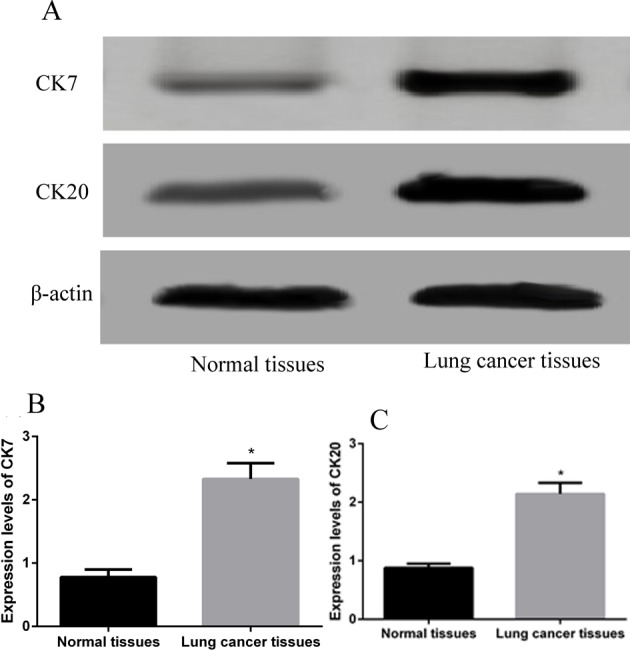
Western blot detection results associated with the protein expressions of CK7 and CK20 in lung cancer and paracancerous normal tissues (**A**) Electrophoresis strip results to reveal the relationship of CK7 and CK20 protein expressions in lung cancer and paracancerous normal tissues, with β-actin as an internal reference. (**B**) CK7 protein expression was obviously increased in lung cancer tissue than that in paracancerous normal tissue. (**C**) CK20 protein expression also showed an increased trend in lung cancer tissue than that in paracancerous normal tissue. *, *P*<0.05, the difference between groups was significant.

Protein expressions of CK7 and CK20 in lung cancer and paracancerous tissues showed that both CK7 and CK20 were also differently expressed, obviously higher trend was found in lung cancer tissues than those in paracancerous normal tissues (both *P*<0.05), which was described in Figure 2. Furthermore, Spearman correlation analysis results showed that there was positive correlation between the expression of CK7 and CK20 (r =0.348, *P*<0.05).

### CK7 and CK20 expressions using immunohistochemistry staining and clinical pathological parameters of lung cancer

**Figure 3:**
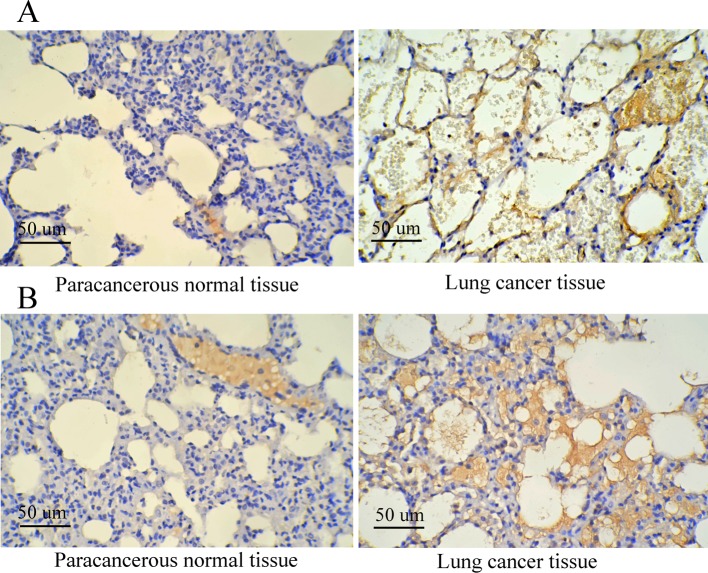
Immunohistochemistry staining results concerning the expression of CK7 and CK20 in lung cancer and paracancerous normal tissues (**A**) Expression of CK7 in lung cancer and paracancerous normal tissues; (**B**) expression of CK20 in lung cancer and paracancerous normal tissues.

**Figure 4:**
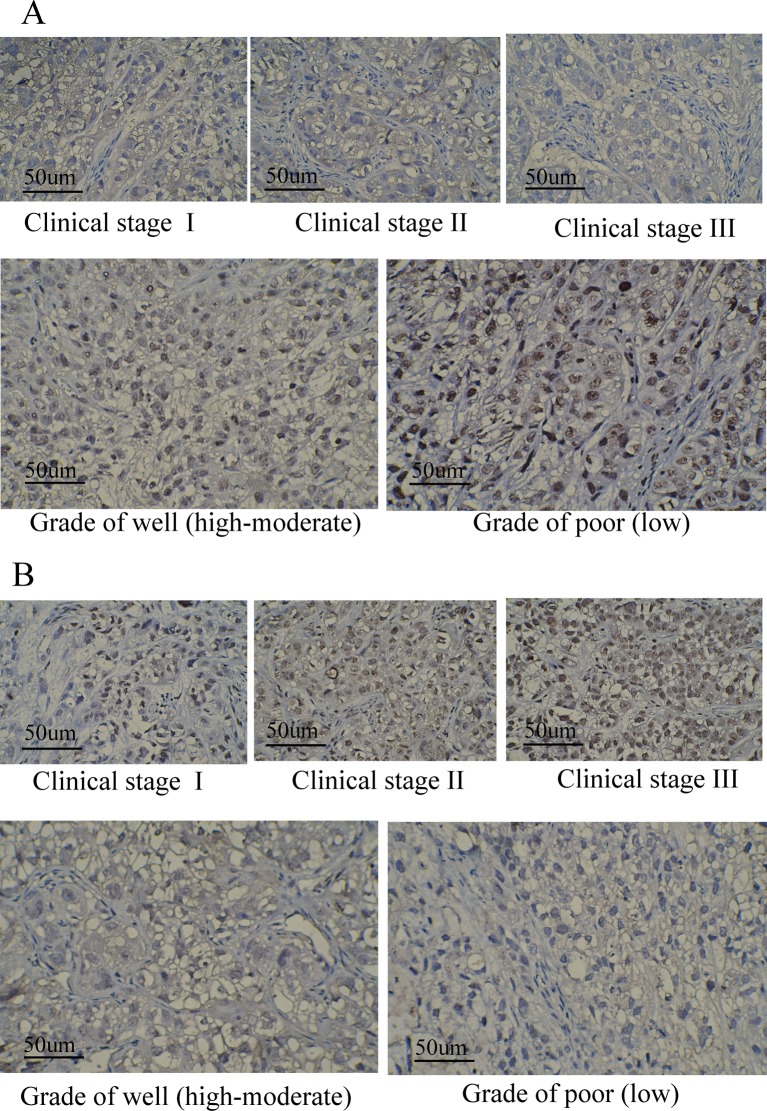
Immunohistochemistry staining results of the expression of CK7 and CK20 in lung cancer according to different clinical stages and grades (**A**) Expression of CK7 in different clinical stages and different grades of lung cancer, clinical stages were classified based on TNM criteria, covering three stages of stages I–III, respectively; different grades were categorized based on different differentiation status, including two grades of well (high-moderate) and poor (low); (**B**) Expression of CK20 in different clinical stages and different grades of lung cancer, clinical stages and grades were divided according to the above-mentioned criteria.

According to the results of immunohistochemistry staining in lung cancer and paracancerous normal tissues, CK20 and CK7 were located in the cytoplasm, and it was found that the cytoplasm or nucleus was brown or yellow, which were shown in Figure 3. As shown in Table 1, positive rates of CK20 and CK7 were 76.74 and 79.07%, respectively. Meanwhile, there were obvious differences regarding the positive expression of CK20 in different T stage, lymph node metastasis, invasion, size, and clinical stage subgroups (all *P*<0.05); but no apparent significance was found regarding sex, age, smoking, and differentiation situation (all *P*>0.05). In addition, significant differences in the positive expression of CK7 were observed in different sex, age, lymph node metastasis, invasion, differentiation subgroups (all *P*<0.05), not including smoking, T stage, size and clinical stage (all *P*>0.05). Both CK20 and CK7 expressions were markedly related to lymph node metastasis and invasion (both *P*<0.05). Meanwhile, CK20 and CK7 expressions in different clinical stage and different grades based on the status of differentiation were also presented in Figure 4, to show the expression of both proteins clearly from the perspective of immunohistochemistry. Both proteins were stained to various degrees in lung cancer tissues at different clinical stages (expressed as different clinical stages) and grades (presented as different differentiation). Besides, under the observation of the positive staining of CK20, the color of immunohistochemistry staining gradually deepened with the deterioration of the clinical stages. At the same time, for CK7, with the deterioration of the clinical grades, the staining color deepened with decreased differentiation degree.

**Table 1:** Demographics and clinical characteristics of patients with lung cancer, as well as the association with the expression of CK20 and CK7

Variables	Expression of CK20 (*n*=258)			Expression of CK7 (*n*=258)		
	Negative expression (*n*=61)	Positive expression (*n*=197)	*P*	Negative expression (*n*=54)	Positive expression (*n*=204)	*P*
Sex						
Male	34	121	0.428	22	133	0.001
Female	27	76		32	71	
Age (years)						
<60	25	71	0.485	30	66	0.002
≥60	36	126		24	138	
Smoking						
No	18	73	0.281	17	74	0.512
Yes	43	124		37	130	
T stage						
T1–2	38	79	0.002	26	71	0.072
T3–4	23	118		28	133	
Lymph node metastasis						
No	36	74	0.003	29	73	0.017
Yes	25	123		25	131	
Invasion						
No	21	113	0.002	28	74	0.037
Yes	40	84		26	130	
Size (cm)						
<3	43	109	0.035	34	118	0.497
≥3	18	88		20	86	
Differentiation						
Well	21	91	0.106	16	96	0.022
Poor	40	106		38	108	
Clinical stage						
Stage I	23	120	0.006	29	114	0.732
Stage II	25	51		15	61	
Stage III	13	26		10	29	

### CK7 and CK20 expressions and prognosis of lung cancer

**Figure 5:**
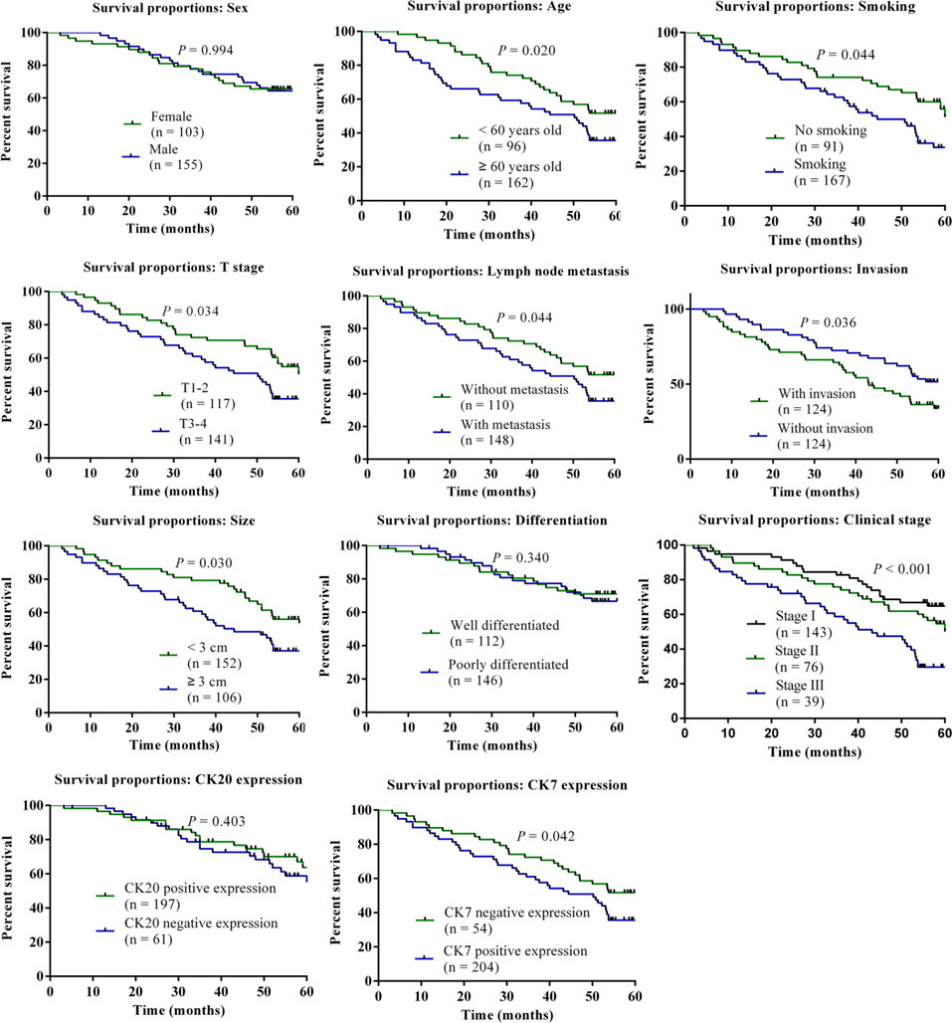
Kaplan–Meier overall survival curve for predictive factors influencing overall survival of lung cancer Examined factors included sex, age, smoking, T stage, lymph node metastasis, invasion, size, differentiation, clinical stage, CK20 and CK7 expression. *P*<0.05 meant that there was a statistical difference in the comparison of different subvariables.

In the enrolled 258 cases with lung cancer treated by surgical treatment, overall survival rate was 60.08% in the follow-up period, with 155 cases of patients who survived during the period of followup. A sum up of 113 of 204 patients with positive CK7 expressions survived with an average survival rate of 55.39%, and 42 of 54 patients with negative CK7 expressions survived with the survival rate of 82.35%. Further, 105 of 197 patients with positive CK20 expressions died with a survival rate of 53.30% and 50 of 61 patients with negative CK20 expressions survived with survival rate of 81.97%; suggesting that positive expression of CK7 and CK20 might have significantly adverse effects on the survival outcomes in patients with lung cancer. Kaplan–Meier method was used for survival analysis of lung cancer related factors, univariate analysis indicated that patients with age greater than 60 years of age, smoking history, T stage, lymph nodes metastasis, invasion, size, and CK7 expressions had relatively poorer survival outcomes, comparison results were statistically significant (all *P*<0.05). The results suggested that effects of age, smoking, T stage, lymph node metastasis, invasion, size, clinical stage, and CK7 expression on the survival of patients were significant. Meanwhile, Figure 5 showed the results of Kaplan–Meier overall survival curve for the above predictive factors influencing overall survival of lung cancer patients.

### COX regression analysis and prognosis

Multivariate analysis by Cox proportional hazard regression model revealed that lymph node metastasis, T stage, and CK7 expression were independent risk factors for prognosis (all *P*<0.05), while age, smoking, invasion, size, and clinical stage had no marked correlation with the survival outcomes of patients with lung cancer (all *P*>0.05) (Table 2).

**Table 2:** Multivariate analysis of overall survival in patients with lung cancer

	B	S.E.M.	Wald	df	Sig.	Exp (B)	95% CI for Exp (B)	
							Lower	Upper
CK7	0.719	0.204	12.369	1	< 0.001	1.487	1.327	1.728
Lymph node metastasis	0.466	0.173	7.263	1	0.007	1.594	1.136	2.238
T stage	0.566	0.180	9.925	1	0.002	1.760	1.238	2.503

Abbreviations: B, regression coefficient; df, degree of freedom; Exp(B), odds ratio; Sig.: significance; Wald, Wald test statistics; 95% CI, 95%confidence interval.

## Discussion

We started the present study to investigate the association of CK7 and CK20 expressions with the clinical parameters and prognosis of lung cancer. Previous researches have revealed that the 5-year survival rate of lung cancer is estimated to below 15%, tumor cells metastasis and high recurrent rate are the major causes for the high death rates in this malignancy [23,24]. In view of increased risk and poor prognostic outcomes of lung cancer, insights into clinical pathological features and survival outcomes of lung cancer has gained in recent decades. A retrospective study was performed to examine the role of CK7 and CK20 expressions in patients with lung cancer. Both qRT-PCR and Western blot revealed that both *CK7* and *CK20* mRNA and protein expressions were increased in lung cancer tissues, meanwhile, there was a positive correlation between them. Furthermore, immunohistochemistry staining results showed that CK20 and CK7 were located in the cytoplasm, the expressions of CK7 and CK20 in lung cancer specimens were positive, further confirmed the results drawn from qRT-PCR and Western blot. Generally, immunohistochemistry staining is a useful method for the identification of protein expressions in tissues and gives a direct and immediate impression of disease condition, in combination with the mRNA and protein expression by qRT-PCR and Western blot would increase the reliability of the results. In the study, positive expression of CK20 and CK7 in tumor tissues and non-carcinoma adjacent tissues of lung cancer might be related to the functional enhancement of intermediate filament synthesis in the cells of these abnormal hyperplastic epithelial tissues, suggesting that the positive expressions of CK7 and CK20 might correlate strongly with the formation of lung cancer and the progression of such cancer. Recent studies have suggested that CK7 and CK20 are positive promoters in the malignant progression with higher expressions in malignant tumor tissues than in normal tissues [25–27].

Previous investigation has indicated that a fragment of CK is a characteristic protein component of epithelial cells, whose abnormal elevation may be potentially associated with the development of various types of lung cancer, therefore, blocking the expression of CK7 and CK20 may be workable for the inhibition of the carcinogenesis process and beneficial for the development of anticancer treatment strategies [28–30]. On basis of the former researches, we came up with a hypothesis in the present study that CK7 and CK20 might be useful predictors for prognosis of lung cancer which has been rarely studied. And associated with the above knowledge, we detected CK7 and CK20 expressions in the collected tissues. Corresponding results suggested that there were obvious differences regarding the increased expression of CK20 in different T stage, lymph node metastasis, invasion, size, and clinical stage subgroups; increased expression of CK7 associated significantly with different ages, lymph node metastasis, invasion, and differentiation. In consistent with predecessors’ observation, our results suggested that CK7 and CK20 might be positively related to the tumor development. Moreover, marked obvious positive CK7 expression were found in patients aged over 60 years old, with smoking history, at T3–4 stages and with lymph node metastasis, invasion, clinical stage, and tumor size over 3 cm compared with patients aged less than 60 years, without smoking history, at T1–2 stages, without lymph node metastasis and invasion, and tumor size less than 3 cm. Besides, former parameters correlated significantly with poorer prognostic outcomes of lung cancer. In view of the above, CK7, age, smoking, lymph node metastasis, invasion, size, and clinical stage might be considered as valuable predicative factors for prognosis of lung cancer patients. Besides, follow-up study showed that the overall survival in patients with positive CK7 and CK20 expressions were significantly lower in comparison with patients with negative CK7 and CK20 expression. Furthermore, COX regression analysis results suggested that CK7 expression, lymph node metastasis, and T stage might be independent factors of poor prognosis of patients with lung cancer, which might be helpful for surgeons and their patients to select adjuvant therapies of solid tumors. Despite its widespread diagnostic use in colorectal and gastric cancers [31–33], little is known about the expression and significance of CK7 in other tumors *in vivo*. Therefore, in the current investigation, following the identification of high expression of CK7 in lung cancer and positive expression of CK7 in different clinical pathological features of lung cancer patients, we further explored the clinical values of CK7 expression in predicting the prognostic outcomes of this cancer associated with its clinical pathological characteristics. CK7 can be specifically expressed in certain cancers, which has been verified to be expressed in lung cancer of the present study; besides, the expression of CK7 is a sign of dedifferentiation, positive cells of CK7 may therefore be relatively lower differentiation cells with high degree of malignancy. In this regard, overexpression of CK7 might serve as an important marker for metastatic lung cancer, we came to our hypothesis that positive CK7 expression in lung cancer might be partially responsible for the poor prognostic outcomes of lung cancer. Meanwhile, we speculated that with the existence of lymph node metastasis, it accelerated cancer cells metastasis, which also correlated with the depth of invasion and differentiation, as well as tumor staging, further became the main contributor to poor prognosis of patients.

However, our study failed to figure out the exact signaling pathway of CK7 and CK20 in lung cancer, and also did not provide further mechanism by which positive CK7 and CK20 were activated in lung cancer. Besides, single detection of CK7 and CK20 without other indicators were not enough to verify our speculation. Another limitation of our study laid in that our study set was in an *in vitro* environment only, which might also lead to possible bias in our results. Although a few investigations in the same field have been published to explore the role of CK7 and CK20 in lung cancer [34], our study focussed on the relationship with the clinical pathological features and the prognostic outcome of involved patients, we also tried to figure out possible prognosis predictive factors, which were obviously different from past research. Future efforts shall pay more attention to the role of CK7 and CK20 and their complex interactions in lung cancer, and to provide more comprehensive studies with lager sample size in an *in vivo* and *in vitro* environment.

In conclusion, we provide stout evidence that CK7 and CK20 are positively expressed in lung cancer; positive expression of CK7 associated with pathological features of lymph node metastasis and T stage may be independent clinical parameters for poor prognosis of lung cancer patients. Future efforts are need to provide more powerful insight into these two proteins associated with *in vivo* experiments and *in vitro* verification.

## Abbreviations

CK: cytokeratin
DAB: diaminobenzidine
ddH2O: double distilled water
GAPDH: Glyceral dehyde-3-phosphate dehydrogenase
OD: optical density
qPCR: quantitative polymerase chain reaction
qRT-PCR: real-time quantitative PCR
